# Thinking about Eating Food Activates Visual Cortex with Reduced Bilateral Cerebellar Activation in Females with Anorexia Nervosa: An fMRI Study

**DOI:** 10.1371/journal.pone.0034000

**Published:** 2012-03-27

**Authors:** Samantha J. Brooks, Owen O'Daly, Rudolf Uher, Hans-Christoph Friederich, Vincent Giampietro, Michael Brammer, Steven C. R. Williams, Helgi B. Schiöth, Janet Treasure, Iain C. Campbell

**Affiliations:** 1 Department of Neuroscience, Uppsala University, Uppsala, Sweden; 2 Department of Neuroimaging, Centre for Neuroimaging Sciences, King's College London Institute of Psychiatry, London, United Kingdom; 3 Department of Psychological Medicine, Section of Eating Disorders, King's College London Institute of Psychiatry, London, United Kingdom; 4 Psychosomatic and General Internal Medicine, Centre for Psychosocial Medicine, Heidelberg, Germany; Royal Holloway, University of London, United Kingdom

## Abstract

**Background:**

Women with anorexia nervosa (AN) have aberrant cognitions about food and altered activity in prefrontal cortical and somatosensory regions to food images. However, differential effects on the brain when thinking about eating food between healthy women and those with AN is unknown.

**Methods:**

Functional magnetic resonance imaging (fMRI) examined neural activation when 42 women thought about eating the food shown in images: 18 with AN (11 RAN, 7 BPAN) and 24 age-matched controls (HC).

**Results:**

Group contrasts between HC and AN revealed reduced activation in AN in the bilateral cerebellar vermis, and increased activation in the right visual cortex. Preliminary comparisons between AN subtypes and healthy controls suggest differences in cortical and limbic regions.

**Conclusions:**

These preliminary data suggest that thinking about eating food shown in images increases visual and prefrontal cortical neural responses in females with AN, which may underlie cognitive biases towards food stimuli and ruminations about controlling food intake. Future studies are needed to explicitly test how thinking about eating activates restraint cognitions, specifically in those with restricting vs. binge-purging AN subtypes.

## Introduction

Anorexia nervosa (AN) is defined by substantial emaciation due to deliberately reduced food intake and it has two subtypes: *restricting (RAN)* and *binge-purging (BPAN)* AN [1]. People with RAN are emaciated due to excessive food restriction. Those with BPAN have sporadic episodes of food consumption, yet are still emaciated due to purging and other compensatory behaviours (e.g. excessive exercise). Commonality between the two subtypes of AN are that they both excessively ruminate on cognitive strategies to control food intake. Functional magnetic resonance imaging (fMRI) is beginning to consistently show that women with AN, in response to appetitive stimuli (e.g. anticipatory food images, food consumption) during the scan have neural responses that indicate excessive ‘top-down’ prefrontal cortical (PFC) combined with reduced ‘bottom-up’ somatosensory appetite-related activation [2], [3], [4], [5]. Therefore, the neural basis of AN may involve interactions between regions that are associated with cognitive control and those with somatosensory responses to food consumption. However, few studies to date have examined how cognitions related to food intake impact on neural activation to food images in those with AN. For example, the neural substrates of cognitions associated with strategies for control may have an overly regulatory role in the restrictive behaviour seen in patients with AN. Furthermore, binge-purging activity observed in some AN cases (e.g. BPAN) may result from a reduced activation of the same regulatory mechanisms, perhaps in the presence of an enhanced motivational drive for food consumption.

Executive function deficits common in those with AN, associated with the PFC, such as excessive asceticism [6], cognitive rigidity/attention to detail [7] and cognitive rumination [8], are likely to be found in interactions between brain regions involved in the cortical Cognitive Control Network (CCN) and appetitive brain regions. The CCN is a network of PFC brain regions linked to executive function (e.g. selective attention, cognitive inhibition, and working memory) that likely regulate subcortical brain regions, which together are involved in appetite regulation (i.e. the striatum, amygdala, hypothalamus cerebellum). Activation of the basal ganglia, particularly the dorsal striatum (for impulsive responses) and the ventral striatum (for wanting and liking) have been associated with appetitive responses in obesity [9] and so one might expect aberrant or reduced activation in the striatum, which combined with irregular activation of somatosensory brain regions, is associated with the experience of anxiety and food restriction in those with AN. The cerebellum, particularly the vermis, is part of the bottom-up appetitive network and has a prominent role in feeding behaviour, particularly the drive to approach appetising stimuli [10], [11], [12]. The interaction between the CCN and bottom-up appetitive regions may determine individual levels of control over the desire for rewarding stimuli [13], [14], [15].

AN is highly comorbid with anxiety disorders [16] and people suffering with AN often experience anxiety, particularly Obsessive Compusive Disorder (OCD) [17] and *alexithymia*, or problems with emotional awareness [18] that are robustly observed in neuropsychological studies [19], [20]. Thus, caution is advised when conducting fMRI studies on those who receive pharmacological intervention for psychiatric disorders (e.g. Selective Serotonin Reuptake Inhibitors [SSRIs] for anxiety) as it can alter the patterns of brain activation observed [21]. Nevertheless, underlying anxiety and *interoceptive awareness* (‘feeling of the body’) is the function of the insular cortex, which has extensive connections between the PFC, somatosensory regions and the cerebellum, such as in states of hunger, emotion and anxiety [22]. A cortico-striatal-insula neural pathway has been implicated in anxiety-related neural responses [23], incorporating the basal ganglia, ACC and insular cortex [24], [25]. However, despite evidence seemingly stacked in favour of the insular cortex being highly involved in the pathology of AN, the actual evidence is still unclear. For example, reduced activation in the insula (in comparison to healthy controls) is reported in response to the receipt of monetary reward and rewarding taste stimuli in people with AN [26], [27], but on the other hand, ratings of anxiety towards high calorie drinks positively correlate with greater insula activation in women with AN [28], while ratings of disgust in response to food stimuli do not correlate [29]. Prominent researchers in the eating disorders field propose that there is a ‘rate-limiting’ defect in insular cortex processing in those with AN, e.g. an inefficient orchestration of cognitive and somatic processing that fosters a balanced cognitive and emotional representation of the body [30], [31].

Against this background, fMRI studies of those who have recovered from AN (e.g. weight gain or reduction of dysfunctional cognitions) show a converse pattern of activation in response to appetitive stimuli, potentially indicative of restored brain function. For example, an increased neural response in the dorsal and ventral striatum, insular cortex and occipital lobe has been reported in comparison to healthy subjects [27], [32]. Another fMRI study using a Region of Interest approach and ingestion of liquid continued to find reduced insular cortex, and striatal activation in comparison to healthy controls [26]. In one fMRI study with food images, comparing those recovered from RAN to healthy controls, an increased medial prefrontal and ACC activation was observed, as well as reduced activation in the inferior parietal lobe, whereas in comparison to those chronically ill, the recovered females had differential frontal and ACC activation [29]. Thus, it might be that bottom-up appetitive neural responses are more prone to normalise following recovery, whereas frontal regions are prone to chronic functional effects specific to risk for developing AN, that are independent of nutritional status (or perhaps exacerbated by starvation). This fits the clinical picture that cognitive dysfunction is one of the main risk factors for AN, appearing before emaciation, and is the last, if ever, to normalise following weight gain [33]. Thus, by asking females to think about eating food during an fMRI paradigm, rather than merely observe food images (as with other previous fMRI studies), we might hone in on the neural mechanisms that are at the core and beginnings of excessive food restriction.

A recent clinical review of neuroimaging studies of those ill with AN [34] reveals that the frontal, parietal and bilateral anterior cingulate cortices are most susceptible to altered neural activation within the subtypes of AN (RAN and BPAN). However, when neural activation is examined in a heterogeneous AN group (e.g. not separating the subtypes) another neural pattern emerges, with the main difference being reduced function/metabolism in the temporal lobe. Therefore, it is also important to examine neural activation in a whole AN group, but also to separate the analyses for the distinct subtypes. Generally, fronto-temporal and somatosensory cortical regions are proposed to interact with basic appetitive brain regions, to predict how the value of a stimulus might affect body state, and also to determine the appetitive needs of the organism, and if these interacting cognitive-arousal systems are awry, deficits in interoceptive awareness and maladjusted reward processing, commonly observed in those with AN [3], might occur. By using a novel fMRI paradigm, adapted from a symptom provocation fMRI study that was first used in those with Obsessive Compulsive Disorder (OCD) [35] for the first time we examine the role of cognitions (or thoughts about eating) in those with AN while they view images of food. We propose to test explicitly, which regions of the CCN interact with appetitive brain regions. In a previous publication we show reduced striatal and insular responses when thinking about eating food, in females with AN compared to females with bulimia nervosa, and that within groups, healthy females have greater appetitive responses to food (vs. non food), whereas females with AN also have an increased right dorsolateral prefrontal cortex (DLPFC) response [36]. Here, we will extend our previous work by comparing females with AN to healthy controls, and conduct preliminary comparison analyses on the subtypes. This should further strengthen our understanding of the neural circuitry of AN, and help to shape treatment that specifically addresses thoughts about eating.

Thus, in order to examine how cognitive systems interact with reward and appetitive systems in females with AN the present novel study (utilising the cognitive factor of thinking about eating the food shown in images) tested the following hypothesis: 1) In women with AN (in comparison to HC), in response to thinking about eating food shown in food (vs. non-food) images, there will be greater activation in regions of the CCN that are linked to cognitive inhibition (e.g. DLPFC) and cognitive evaluation of reward expectancy (e.g. ACC) and reduced activation in regions associated with appetitive and somatosensory impulsive responses (e.g. dorsal striatum, insular cortex, cerebellar vermis). 2) Women with AN (in comparison to HC) will report higher levels of anxiety when viewing food images and their levels of anxiety will correlate with CCN and insular cortex activation.

As a second exploratory step, we have separated our main cohort of AN females into two smaller groups of women with RAN (n = 11) and BPAN (n = 7) and hypothesise that food stimuli will elicit greater activation in the CCN in RAN, while in those with BPAN there will be greater activation in appetitive and somatosensory regions.

## Methods

### Ethics statement

This study was approved by the South London and Maudsley (SLaM) NHS Trust Ethics Committee, study number: 297/02. Additionally, the study adhered to the guidelines as set out in the Declaration of Helsinki. Written informed consent was required from all participants, as approved by the SLaM ethics committee, and they were reimbursed for their participation.

### Participants

42 right-handed females aged 16–50 years participated in the study, 24 healthy controls (HC) who did not have a history of psychiatric disorder and who were within the normal BMI, and 18 with a current DSM-IV diagnosis of AN. The AN group included 11 with a diagnosis of *restricting* AN (RAN) and 7 with *binge-purging* AN (BPAN). Women with AN were receiving inpatient treatment at the Bethlem Royal Hospital, South London and Maudsley (SLaM) NHS Trust. Diagnosis of AN (and subtype) was primarily made by a consultant psychiatrist, and confirmed using the Structured Clinical Interview for DSM-IV [37]. All participants completed self-report questionnaires (see below) before the experiment. The HC volunteers were recruited from college students who responded to an advertisement. The women were matched for age and IQ and were instructed to eat lunch, but not to eat or drink anything containing caffeine or other stimulants for two hours, or to drink alcohol for twenty-four hours prior to the experiment. Exclusion criteria were a history of head trauma, hearing or visual impairments, neurological disease, metallic implants, claustrophobia and psychotropic medication other than selective serotonin reuptake inhibitors (SSRIs). Since it has been shown that pharmacological intervention can significantly alter the pattern of neural activation observed in fMRI studies [21], we conducted post-hoc t-test analyses (on brain regions that were significantly different between AN and HC) between those AN patients who were taking SSRI medication and those who were not (see results section). This was done to ensure that the brain activation we observed was due to having AN and not due to pharmacological effects. Participants gave written informed consent, as approved by the SLaM NHS Trust Ethics Committee, and were paid £30 for their participation. Due to technical issues, and one participant feeling claustrophobic and withdrawing from the experiment, 5 women with AN (3 with RAN and 2 with BPAN) and 3 HC women were excluded: thus, 18 women with AN (11 with RAN, 7 with BPAN) and 24 HC women contributed to the analysis.

### Stimuli

72 colour photographs of high and low calorie, sweet and savoury food (e.g. hamburgers, chocolate brownies, sandwiches) were presented on white plates and a blue background in random order: these were created by the authors. The control condition was made up of 72 colour photographs of non-food items (e.g. a stapler, yellow clothes pegs) on white plates and a blue background (created by the authors). Food and non-food items were selected and matched according to colour and visual structure (e.g. yellow clothes pegs on a plate matched with chips on a plate). All the images are available on request [36]. **See Supplementary Table S1 for the list of images used**.

### Questionnaire Measures

#### The Eating Disorders Examination – Questionnaire ([EDE-Q,[38])])

This is a 36-item measure of dysfunctional behaviour and cognitions related to eating, with sub-scales: eating concern, shape concern, weight concern and restrained eating, together with a global eating disorder score. Questions are scored between 0–6, with a high score indicating greater eating disorder pathology.

#### The Hospital Anxiety and Depression Scale (HADS,[39])

This is a 14-item self-report measure with 7 related to anxiety and 7 related to depression. Individual questions are scored on a 4-point scale, with higher scores indicating greater anxiety or depression.

#### The Structured Clinical Interview for Diagnosis-Researcher Version (SCID-R) [37]

This structured interview is used to diagnose AN, for general screening, and to obtain demographic information. Duration of illness is the time between diagnosis of AN and the time of the scan. It is noted that AN symptoms are most likely present before the formal diagnosis, but this measure gives a systematic score of illness duration.

### Procedure

We conducted the scans between 1.30 and 4 pm. Images were presented on a rear-projection screen and viewed through a double-mirror periscope attached to the headcoil. Images (food versus non-food) were presented during the same scanning period. An AB block-design of 6 blocks for the experimental condition (food images [A]) and 6 blocks for the control condition (non-food images [B]) was used: blocks were alternated between the experimental and control conditions. Each block comprised of 12 images presented without a break in between, for 3 seconds, i.e. images for each condition were presented continuously for 36 seconds. At the beginning of each block a ‘partially silent’ period of 8 seconds, and another partial silent period for 8 seconds at the end of each block (where no data was acquired and the Echo Planar Image [EPI] readout was disabled) was used to present audio stimuli and to obtain verbal responses from each participant. During these partially silent periods, slice selection, Radio Frequency (RF) pulse and gradients continued in order to maintain the MR signal in a steady state and to allow data collection to follow a comparable strength across subsequent volumes. Audio stimuli was pre-recorded by an adult female, asking participants to a) imagine eating the food in the images, and b) imagine using the non food items. For each instruction, 4 separate but semantically similar phrases were given via headphones. In the second partially silent period (at the end of each block), participants were asked to rate how anxious they felt on a scale of 0–10: participants responded verbally. The duration of each block was 52 seconds (36 seconds of stimuli and two 8 second periods of ‘partial silence’), repeated 12 times (food versus non-food): total duration of the presentation of food versus non-food images was therefore 10.4 minutes.

### Image acquisition

All fMRI data was acquired on a GE Signa 1.5 Tesla scanner (GE Medical Systems, Milwaukee, Wisconsin) housed at the Centre for Neuroimaging Science (CNS) Building, King's College London, Denmark Hill Campus. T2* - weighted images depicting Blood Oxygen Level Dependent (BOLD) contrast were acquired with a TR of 4 seconds (repetition time) with an in-plane resolution of 3.75 mm×3.75 mm. The echo time was 40 msec and the flip angle was 90°. Whole brain coverage was acquired in 43 slices (slice thickness 3 mm, interslice gap 0.3 mm). Fifty-four T2*-weighted whole brain volumes were acquired in each of the two conditions in both experiments.

### Data analysis

Data was analysed with the XBAM software developed at the Institute of Psychiatry [40], [41], [42]. Analysis based on 3D clusters provides a more powerful measure of brain activation that take place over a number of contiguous voxels, as opposed to statistics computed using information from a single voxel [43] and so we used a cluster-based approach. Parametric distribution is not known for cluster mass, and also group fMRI data is not often normally distributed [44] and so a non-parametric, permuation-based analysis was used in this case, to assess statistical significance. The parametric approach available within SPM software (Institute of Neurology, UCL, London: www.fil.ucl.ac.uk/spm) limits to voxel level statistics, and may not be as sensitive as XBAM for detecting neural differences in response to emotional stimuli [45] and so the XBAM software was deemed to be the best statistical approach in this case. Following motion correction, the estimated BOLD effect was modelled by two Poisson functions with haemodynamic delays of 4 and 8 seconds. The least-squares model of the weighted sum of these two functions was compared with the signal in each voxel to obtain a goodness of fit statistic. The distribution of this statistic under the null hypothesis was calculated by wavelet-based resampling of the time series and refitting the models to the resampled data. Active versus control conditions were analysed in Whole Brain generic group activation maps, and these were constructed by mapping the observed and randomised test statistics into standard space, and then by calculating and testing median activation maps. Use of medians prevented the interfering effects of outliers. Between-group differences were generated using a local 3D cluster analysis: this prevented large brain regions, such as the visual cortex from biasing the randomised null distribution and thus enabled the detection of smaller regional neural activations. Cluster-level inference with data randomisation between groups was used to determine the sampling distribution of group differences under the null hypothesis. Voxel- and cluster-wise corrections (using a more stringent False Discovery Rate [FDR] threshold correction at p = 0.01 than the default p = 0.05) were applied (due to small group numbers) to ensure that data was significant at the rate of one or less false positive 3D cluster per brain.

Self-reported data for within and between-subject differences were calculated using repeated measures analysis of variance (ANOVAs) and post-hoc t-tests to confirm the direction of the differences. Examination of associations between continuous variables was calculated using the Spearman's rank non-parametric correlation coefficient (Spearman's Rho = . Associations were deemed significant if correlations met the p-value threshold after Bonferroni correction.

## Results

### Sample characteristics

Preliminary data from 18 women with AN (11 RAN, 7 BPAN) and 24 HC were analysed. Due to time and financial constraints of the study we were unable to increase the subgroup numbers to 12 to meet the minimum p = 0.05 threshold for eighty percent power per voxel [46]. However, we emphasise that the AN subgroup data is exploratory and designed to generate robust hypotheses for future studies. To increase the power of the data we report, we applied stringent voxel- and cluster-wise False Discovery Rate (FDR) correction to ensure that, despite the small sample sizes only highly significant data are reported, and we give effect size data in the table. The main focus of this paper is on the AN v HC contrasts. We present AN and HC group data in our previous publication, but the difference between this paper and the previous paper is that in the previous paper we instead focus on AN vs. bulimia nervosa (BN) and HC v BN neural activation contrasts [36]. See **Table 1** **for demographic and behavioural data**.

**Table 1:** Demographic characteristics and self-report measures: demonstrating the mean values, standard deviations, and differences in scores between women with anorexia nervosa, women with restricting anorexia nervosa, women with binge-purging anorexia nervosa and healthy control women.

	Means, S.D.				Contrast Effect Sizes (cohen's *d*)			
	AN(n = 18)	RAN(n = 11)	BPAN(n = 7)	HC(n = 24)	ANvHC	RANvHC	BPANvHC	RANvBPAN
**Age**, years	26(6.8)	26(7.2)	25(6.6)	26(9.5)	0	0.02	0.07	0.06
**BMI**, kg/m^2^	15.7(1.2)	15.2(1.2)	16.4(0.6)	21.7(2.4)	3.1	2.93	2.66	1.22
**Education**, years	8(3)	8(4)	8(3)	12(6.5)	0.76	0.7	0.76	0.14
**Duration of ED**, years	7.2(4.0)	9(7.4)	9(6.1)	-	-	-	-	0.01
**SSRI medication** (No., %)	10(55.5)	4(36)	6(86)	-	-	-	-	0.04
**EDE-Q (0–6)**								
- restrained eating	2.6(1.7)	2.1(1.6)	5.5(1.8)	0.8(1.0)	1.81	1.1	1.2	0.92
**EDE-Q No. of binges in a month:(Q8, 0–6)**	0.9(1.78)	-	2(3)	-	-	-	-	-
**EDE-Q No. of vomits in a month: (Q22)**	2.8 (7.28)	-	7(10)	-	-	-	-	-
**HADS- Anxiety (0–21)**	13.6(3.6)	12(9)	17(3)	4.4(2.7)	4.19	2.7	2.8	1.14
**HADS- Depression (0–21)**	13.6(3.7)	8(5)	12(9)	1.6(1.7)	4.5	2.9	2.7	0.88
**Psychiatric co-morbidity (No., %)**	14(77.7)	8(78)	6(71)	-	-	-	-	0.04
- Depressive disorders (No., %)	3(16.6)	2(18)	1(14)	-	-	-	-	0.06
- Anxiety disorders (No., %)	6(33.3)	8(27)	2(29)	-	-	-	-	0.04
- Depression & Anxiety (No., %)	5(27.7)	8(27)	2(29)	-	-	-	-	0.04
**Mood (0–10)**	4.2(2.2)	4.47(2.3)	8.91(2.2)	6.4(1.7)	0.28	1.05	1.43	2.08
**Food anxiety (0–10)**	6.9(1.8)	6.98(2.1)	7.66(2.0)	2.4(2.0)	0.24	2.32	2.72	0.35
**Non-food anxiety (0–10)**	1.8(0.9)	1.44(0.78)	2.68(0.48)	1.8(1.7)	0.86	0.25	0.59	1.93

Values expressed as mean, standard deviations in parentheses (S.D.); ABBREVIATIONS: AN = Anorexia Nervosa; RAN = Restricting Anorexia Nervosa; BPAN = Binge Purge Anorexia Nervosa; HC = Healthy Control; BMI = Body Mass Index; ED = Eating Disorder; anx = anxiety measured during the scan as a self-report verbal response where 0 = least anxious and 10 = most anxious; SSRI = Selective Serotonin Reuptake Inhibitor; EDE-Q = Eating Disorder Examination Questionnaire, 0–6 scale for subscale scores where 0 is least severe, 6 is most severe, EDE-Q (Q8) number of binges in one month, scale 0 = no days, 1 = 1–5 days, 2 = 6–12 days, 3 = 13–15 days, 4 = 16–22 days, 5 = 23–27 days, 6 = everyday, EDE-Q (Q22) number of vomits in month = absolute number of vomits in one month, HADS = Hospital Anxiety and Depression Scale, 0–21 scale where 0 is least severe and 21 is most severe for trait anxiety and depression as subscales; effect sizes for the contrasts are calculated using Cohen's *d*.

As expected women with AN had a significantly lower BMI than HC (p<0.001), and women with RAN had significantly lower BMI than those with BPAN (p<0.01), although both subgroups of AN were below the 17.5 BMI cut-off criteria for AN as currently specified by the DSM-IV. The mean age was 26 (6.8) in the AN group and 26 (9.5) in the HC group, with no significant difference between groups. Women with AN had significantly less years of formal education than the HC group (p<0.05), and scored significantly higher on the EDE-Q measure of restraint than the HC women (p<0.01). Women with BPAN scored significantly higher than the RAN on the EDE-Q restraint scale (p<0.01), perhaps a reflection of the desire to restrain rather than actual restraint. Women with AN reported a significantly lower mood before the scan than the HC women (p<0.01), however, women with BPAN reported a higher mood than both RAN and HC groups (p<0.001). As expected, women who were diagnosed with RAN reported no bingeing or purging behaviour, whereas the BPAN group did, which is consistent with the diagnostic criteria for the subtypes. Duration of illness was not significantly different between the subtypes of AN.

### Subjective anxiety ratings prior to and during the scan

Women with BPAN reported significantly higher levels of anxiety prior to the scan (as measured by HADS) than the women with RAN (p<0.001), but were comparably depressed prior to the scan (as measured by HADS). As expected, women with AN were significantly more anxious and depressed than the HC group (p<0.001). With regard to the anxiety ratings during the scan, the subtypes of AN were comparably anxious when thinking about eating the food shown in the food images, and all women with AN were significantly more anxious than the HC group (p<0.001). Women with RAN were comparably anxious in response to the non-food images as the HC group; however, the women with BPAN were significantly more anxious to the non-food images than the RAN and HC groups (p<0.01).

### fMRI data: Within-group maps

#### Food versus non-food images

These data are shown in **Table 2**, and data for healthy controls and the total AN group are reported in our previous publication [36]. In the subgroups of AN, we found that in the RAN group (n = 11), food images significantly increased activation in cerebellar vermis (x = −25, y = −66, z = −16), left visual cortex (x = −14, y = −83, z = −7), right DLPFC (x = 40, y = 5, z = 24), and in the medial prefrontal cortex (x = 0, y = 42, z = 40). In the BPAN group (n = 7), food images significantly increased activation in the left cerebellar vermis (x = −4, y = −56, z = −27), right cerebellar vermis (x = 25, y = −62, z = −14) and right inferior temporal gyrus (x = 22, y = −6, z = −44).

**Table 2:** Within-group brain activation to food versus non-food images in women with restricting anorexia nervosa and women with binge-purging anorexia nervosa.

Brain regions	BA	Laterality	x	y	z	Cluster Size (voxels)	Cluster P
*Food>Non-Food*							
**RAN (n = 11)**							
Cerebellar Vermis	–	L	−25	−66	−16	41	0.001
Visual Cortex	18	L	−14	−83	−7	50	0.001
DLPFC	9	R	40	5	24	66	0.002
Medial PFC	8	—	0	42	40	179	0.0006
**BPAN (n = 7)**							
Cerebellar Vermis	—	L	−4	−56	−27	111	0.0008
Cerebellar Vermis	—	R	25	−62	−14	389	0.0002
Inferior Temporal Gyrus	20	R	22	−6	−44	124	0.0009

*Talairach Coordinates = x, Saggital plane, y, Coronal plane, z, Axial plane; Cluster size in voxels, each voxel equals 3.75 mm×3.75 mm×3 mm; Cluster p = cluster probability corrected at the level of one false positive or less. ABBREVIATIONS: BA = Brodmann's Area; Laterality = L, Left, R, Right; p = probability; AN = Anorexia Nervosa, RAN = Restricting Anorexia Nervosa, BPAN = Binge Purge Anorexia Nervosa, HC = Healthy Controls; DLPFC = Dorsolateral Prefrontal Cortex, SMA = Supplementary Motor Area. Note. Healthy control and total AN group data published in Brooks et al., (2011), PLoS One.*

### fMRI data: Between-group maps

#### Activation to food images compared to non-food images. Data are shown in Figs. 1 and 2, and Table 3

**Figure 1:**
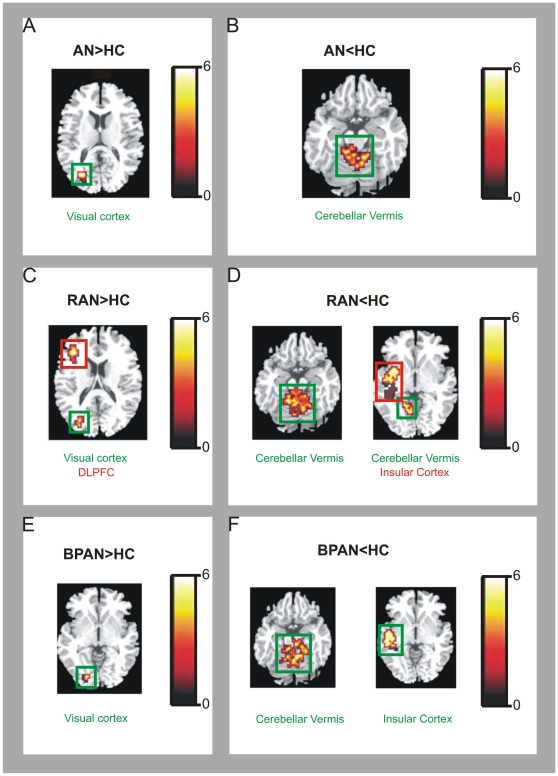
Between-group map activation comparing the food>non-food activation in women with anorexia nervosa versus healthy controls; restricting anorexia versus healthy controls; binge-purging anorexia nervosa versus healthy controls. T-value bar illustrates t-value scores represented by cluster on the brain map. Greater activation in: (**A**) women with anorexia nervosa (AN) compared to healthy controls (HC) in the right visual cortex; (**B**) healthy controls (HC) compared to AN in the bilateral cerebellum; (**C**) women with restricting anorexia nervosa (RAN) compared to HC in the right dorsolateral prefrontal cortex (DLPFC) and right visual cortex; (**D**) HC compared to RAN in the right cerebellum and right insular cortex; (**E**) women with binge-purge anorexia nervosa (BPAN) compared to HC in the right visual cortex; (**F**) HC compared to BPAN in the left cerebellum and right insular cortex.

**Figure 2:**
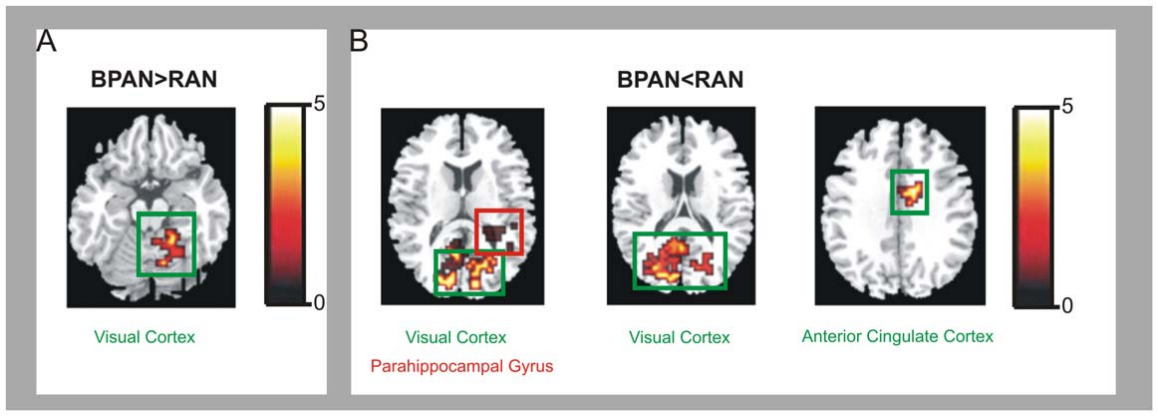
Between-group map activation comparing the food>non-food activation in women with restricting anorexia nervosa versus women with binge-purging anorexia nervosa. T-bar illustrates t-value scores represented by cluster on the brain map. Greater activation in: (**A**) women with binge-purge anorexia nervosa (BPAN) compared to women with restricting anorexia nervosa (RAN) in the left cerebellum; (**B**) women with restricting anorexia nervosa (RAN) compared to BPAN in the bilateral visual cortex, parahippocampal gyrus (PHG) and anterior cingulate cortex (ACC).

**Table 3:** Between group contrast activation to food images in women with anorexia nervosa, women with restricting anorexia nervosa, women with binge-purging anorexia nervosa and healthy control women.

Brain Regions	BA	Laterality	x	y	z	Cluster size (voxels)	Cluster-p
*Food>Non-Food*							
**AN>HC**							
Visual Cortex	18	R	29	−67	15	22	0.0009
**HC>AN**							
Cerebellar Vermis	—	R	14	−33	−15	22	0.0005
Cerebellar Vermis	—	L	−7	−44	−17	30	0.00007
**RAN>HC**							
Visual Cortex	19	R	29	−75	20	23	0.002
DLPFC	46	R	40	37	15	29	0.007
**HC>RAN**							
Cerebellar Vermis	—	L	18	−41	−17	123	0.0003
Insular Cortex	13	R	43	−25	1	149	0.0003
**BPAN>HC**							
Visual Cortex	18	R	18	−76	−2	18	0.0007
**HC>BPAN**							
Cerebellar Vermis	—	L	−6	−34	−23	123	0.0003
Insular Cortex	13	R	41	−21	7	149	0.0003
**RAN>BPAN**							
Visual Cortex	19	R	22	−65	−3	29	0.005
Visual Cortex	18	L	−11	−78	17	71	0.0007
PHG	27	L	−18	−32	2	28	0.009
ACC	23	L	−4	−18	32	24	0.005
**BPAN>RAN**							
Visual Cortex	19	L	−15	−62	−3	10	0.006
*Food>Non-Food*							
**AN>HC**							

*Talairach Coordinates = x, Saggital plane, y, Coronal plane, z, Axial plane; Cluster size in voxels, each voxel equals 3.75 mm×3.75 mm×3 mm; Cluster p = cluster probability corrected at the level of one false positive or less. ABBREVIATIONS: BA = Brodmann's Area; Laterality = L, Left, R, Right; p = probability; AN = Anorexia Nervosa, RAN = Restricting Anorexia Nervosa, BPAN = Binge Purge Anorexia Nervosa, HC = Healthy Controls; DLPFC = Dorsolateral Prefrontal Cortex; PHG = Parahippocampal Gyrus; ACC = Anterior Cingulate Cortex.*

In comparison to the HC group, when thinking about eating food shown in the images, the AN group showed increased activation in the right visual cortex (x = 29, y = −67, z = 15), but relatively reduced activation in the right cerebellar vermis (x = 14, y = −33, z = −15) and left cerebellar vermis (x = −7, y = −44, z = −17).

In preliminary analyses, the RAN group compared to the HC group showed increased activation in the right visual cortex (x = 29, y = −75, z = 20) and right DLPFC (x = 40, y = 37, z = 15) and reduced activation in the left cerebellar vermis (x = −18, y = −41, z = −17) and right insular cortex (x = 43, y = −25, z = 1). Compared to the HC group, the BPAN group showed significantly increased activation in the right visual cortex (x = 18, y = −76, z = −2), but relatively reduced activation in the left cerebellar vermis (x = −6, y = −34, z = −23) and right insular cortex (x = 41, y = −21, z = 7). Finally, in the RAN group compared to the BPAN group, there was relatively increased activation in the right visual cortex (x = 22, y = −65, z = −3), left visual cortex (x = −11, y = −78, z = 17), left parahippocampal gyrus (x = −18, y = −32, z = 2) and left anterior cingulate cortex (x = −4, y = −18, z = 32), but reduced activation in the left visual cortex (x = −15, y = −62, z = −3).

### Correlation Analysis with behavioral data and brain activation

In all groups, no global brain correlations survived Bonferroni corrections.

### fMRI data: effects of SSRIs

In the AN group, 4/11 women with RAN and 6/7 women with BPAN, were taking SSRI medication (which might alter neural activation [21]. Independent t-tests were run to check for differences: no significant differences were found in any neural activation to food or aversive stimuli between women who were taking SSRI medication and those who were not. However, we are aware that all fMRI analyses presented in this paper are limited by power due to the small group numbers. Thus, although much support is gleaned by previous studies, caution must be exercised when interpreting the data presented here. Our intention is to provide preliminary exploratory data in order to generate a platform for future hypothesis testing.

## Discussion

This fMRI study uniquely demonstrated how thinking about eating food presented in images activates differential neural responses between healthy women and those with a current diagnosis of anorexia nervosa (AN). Additionally, we conducted a preliminary analysis of the differential neural responses between the subtypes of AN who have varying levels of appetite restraint. This study progresses our recent fMRI findings that compared women with AN with those who had bulimia nervosa (BN) [47], by focusing only on the differential effects of thinking about eating food between healthy women and those with AN (and the subtypes). Our main finding was that in the total AN group, thinking about eating food corresponded to a reduced activation in the cerebellar vermis and increased activation in the visual cortex. FMRI studies of those with AN showing food stimuli also report reduced activation in the cerebellum [48] and this also accords with reports that the cerebellum, particularly the vermis, has a prominent role in feeding behaviour [10], [11], [12]. It is likely that asking females with AN to think about eating food does not activate appetitive regions of the brain in the same way as healthy females, and it is likely that there will be a reduced appetitive response in the brain, given that one of the core symptoms of this eating disorder is restraint of appetite.

Our strongest result in terms of cluster size, was observed in preliminary analyses comparing the subtypes of AN separately with healthy controls, yielding a reduced activation in the right insular cortex in AN. The insular cortex, particularly the anterior insular cortex (AIC) is associated with subjective feelings of the body, or *interoceptive awareness* and generally with all types of emotional feelings [49], and a recent fMRI study has also shown reduced AIC activation in response to rewarding stimuli in people with AN [36]. Activation of the insular cortex in response to food images in healthy controls might reflect an interoceptive awareness of appetitive responses. However, in women with AN, appetitive responses to food images are likely to be in conflict with the desire to be thin, and to activate anxiogenic concerns about shape, weight and eating. It has been suggested that disruption to insular cortex activation in those with AN might reflect a rate-limiting defect, that is, a failure to efficiently process information from appetitive brain regions and to effectively orchestrate with higher-order cognitions [2], [30], [31]. A rate-limiting defect in the insular cortex likely underlies a proneness for anxiety [24], which often precedes and is highly comorbid with eating disorders [17]. Thus, in females with AN, activation of brain regions involved in the Cognitive Control Network (CCN), such as the DLPFC and the ACC, may be associated with attempts to cognitively control and/or disrupt somatic responses [50] that is the basis of an anxiogenic interoceptive awareness of appetitive stimulation, via interaction with the insular cortex [2], [24], [30], [31], [51].

It is clear from the literature that using body image stimuli, as opposed to food images during fMRI activates different patterns of neural activation in those with AN. Recent studies showing images of bodies (e.g. own, others, drawn figures) report reduced activation in the superior temporal lobe, visual cortex, ACC and frontal regions, as well as increased insula and premotor cortex activation [52], [53], [54]. Therefore, it is likely that body image distortions arise from a different neural pattern to the neural mechanisms underlying restriction of appetite, given that we and others using food stimuli observe increased activation in frontal, ACC and visual regions, but reduced activation in the striatum, cerebellum, insular cortex e.g. [48], [55], [56], [57], although we failed to observe increased amygdala and ACC activation as in some studies e.g. [56], [58]. Furthermore, some fMRI studies demonstrate that hunger and satiety can alter the response to food stimuli in those with AN, for example, reduced visual cortex activation when hungry but greater visual and lateral prefrontal activation when satiated [57], which compares to our results and may demonstrate that females with AN were satiated after eating lunch.

Greater visual cortex activation in the women with AN may be associated with cognitive biases [59] linked with thoughts about consuming the food shown in the images and strategies to cognitively restrain appetite. There is some evidence that the visual cortex is modulated by the dorsolateral prefrontal cortex (DLPFC), via increased attention resources, and the combined activation of these regions are strongly linked to object recognition [60]. Given that we found, in comparison to healthy controls, increased DLPFC and visual cortex activation in just over half the group of AN participants who were sub-classified as *restricting* AN (and not when combining with, or separately analysing the *binge-purging* AN group), it seems a plausible preliminary conclusion to suggest that excessive appetite suppression and cognitive biases for food stimuli in those with RAN are linked to DLPFC function. It could be that combining the subtypes of AN during fMRI analysis prevents DLPFC activation from being observed, given their varying levels of control over appetite. This is a particularly attractive explanation in line with a recent review of fMRI studies in AN that reported a strong association between RAN and DLPFC function [34]. Furthermore, mounting evidence, in both healthy and disordered eating behaviour, implicates the DLPFC in the cognitive control of appetite [61], particularly in females [62], [63], and artificial stimulation of the DLPFC reduces the experience of craving for food in those who are prone to binge eat [64]. Finally, there is evidence for lateralization of DLPFC-driven cognitive control: the left DLPFC activating to anticipation of conflict, the right to immediate, impulsive conflict [65], and here we found increased right DLPFC activation in RAN, suggesting that thinking about eating while looking at pictures of food causes immediate conflict with desires to remain thin.

Preliminary findings when comparing *restricting* with *binge purging* AN also revealed greater activation of the anterior cingulate cortex (ACC) and the parahippocampal gyrus. The ACC lies caudal-medial to the DLPFC, and is associated with orchestrating top-down cognitive and bottom-up arousal (e.g. appetitive) responses, in line with current predictions about desired and likely future rewards [66], in order to make adjustments for effective cognitive control via interaction with the DLPFC [67]. It is plausible that there is more conflict between cognitive strategies and appetitive responses in those with RAN, given that they are perhaps ruminating on restraint cognitions to a greater degree than those who temporarily relinquish their cognitive control during sporadic binge eating, and that appetitive responses interfere with these cognitions. However, it could also be that predictions are computed via ACC activation in those with BPAN, reflecting a greater impingement of appetitive neural circuitry on cognitive restraint, as seen in some fMRI studies of those who are prone to binge eating [68]. Observing a greater parahippocampal response in RAN compared to BPAN might reflect that the former group are engaging in more ruminations about previous experiences of eating food, e.g. [69]. Conversely, it might be that a general lack of appetitive behaviour in those with RAN leads to a downregulation of parahippocampal gray matter volume, e.g [55], causing this region to be hyper-stimulated when explicitly thinking about eating food. However, in our current small preliminary samples of women with RAN and BPAN, it is difficult to ascertain the true nature of the relationship between appetitive processes and cognitive restraint, and so a comparison with larger subgroup groups of AN is needed.

According to a recent clinical review of neuroimaging studies in AN, summarising findings from fMRI and other methods (e.g. using Positron Emission Tomography, Single Photon Emission Tomography) [34], three main regions in those with *restricting* AN show the most consistent dysfunctional activation: the frontal cortex (particularly the DLPFC) [70], [71], [72], [73], [74], [75], hypo-responsiveness in the left inferior parietal lobule [70], [75] and overall dysfunction in the bilateral anterior cingulate cortex [72], [73], [74], [76]. In those with *binge purging* AN there appears to be a similar pattern of neural dysfunction, in the frontal lobe [71], [74], [77], parietal cortex [71], [77] and bilateral anterior cingulate dysfunction [74], [78]. However, when examining neural dysfunction in AN as a whole group (without separately analysing the subtypes) a different neural profile emerges. Instead, it appears that core neural pathology in those with AN in general, includes reduced function/metabolism in the temporal lobe [47], [79], [80], [81] seemingly improving following recovery [82], [83], [84]. Moreover, in response to food stimuli, a left-dominant reduction in the temporal and parietal lobes is prevalent in people with AN [28], [29], [48], [85]. Increased activity in the fusiform gyrus and amygdala are also commonly observed [28], [82], [83], [84], [85]. Furthermore, dorsal striatum hypo-activation in AN regardless of subtype has also been reported by functional neuroimaging studies [71], [82], [83]. Thus, in conjunction with the main findings of previous neuroimaging studies, we find that thinking about eating food is associated with neural dysfunction in the visual cortex, DLPFC and ACC, and that a different neural signature is observed when separating the subtypes of AN, prompting the need for further research in to the subtypes.

In the present study, we used a symptom-provocation fMRI paradigm that has been used in investigations of Obsessive Compulsive Disorder (OCD) [35], an anxiety disorder that is highly comorbid with AN [86] . In the previous study of those with OCD, participants were cognitively engaged when they were shown anxiogenic pictures related to their disorder. They reported being anxious and showed an increased medial and dorsal PFC activation. In comparison, during our scanning period, we found that cognitively engaging participants while presenting food images (by asking them to think about eating the food) induced significantly higher levels of anxiety in women with AN compared to healthy controls. In addition, the RAN group showed a prefrontal response to the food images. Using instructions to cognitively engage the participants is a methodological variation from other fMRI studies that merely present appetitive stimuli (images of food, drink, and taste in the mouth) for passive engagement (e.g. not explicitly instructing to cognitively engage) to women with AN [26], [28], [29], [48], [57]. It is of note that these previous studies did not observe an increased DLPFC response to the appetitive stimuli. Thus, activation of the DLPFC may reflect a specific recruitment of anxiety-related cognitions in women with AN in relation to food (e.g. concerns about shape, weight and eating, striving for thinness), that may drive an inhibition of the insular cortex and other brain regions linked to appetitive responses (e.g. the cerebellum) that arise when thinking about eating food shown in images.

There are some caveats to the explanations above, and some limitations in this study. We did not find amygdala or OFC activation to food images in women with AN as in other studies [28], [29], [48], [87], [88], [89]. However, the size of the amygdala, combined with the reduced power of the study due to small sample sizes, and the heterogeneity of the AN group as a whole may account for this. However, again these differences could be due to a greater cognitive component during this paradigm in comparison to other studies. It is a fact that our subgroup numbers are small. However, we emphasise the preliminary nature of this unique fMRI study utilising cognitive engagement in the images in line with our previously published fMRI data [36]. In an attempt to counteract some of the limitations of small group numbers we used stringent voxel- and cluster-wise False Discovery Rate (FDR) threshold correction. Additionally, some women with AN were taking SSRI medication, but independent t-tests showed no significant differences in neural activation between those who were and were not taking SSRI. Also, it must be considered that since the females with AN were currently ill, the neural activation we observe could be due to malnutrition effects, and future fMRI studies should add covariates for gray matter volume [3]. Furthermore, we only used high calorie food images, and did not compare neural and cognitive responses to low calorie images, nor were food preferences assessed, which could have caused differential activation. Also, we did not collect data to ensure that all healthy participants had, as instructed, eaten lunch, although all females with AN were collected from the hospital after eating lunch; nor did we collect data on IQ or duration of impatient care (although we did collect duration of illness data). Lastly, we did not explicitly test cognitive engagement with the images: however, increased visual cortex activation (in the food versus non-food individual group contrasts) suggests visual engagement in the images during the experiment.

The data from this novel preliminary study progresses the field and can provide the following tentative conclusions that need further testing. Women with AN have reduced appetitive and somatosensory neural responses to food images when explicitly thinking about eating food shown in images, but the subtypes are differentiated by increased DLPFC-ACC and reduced cerebellar vermis activation. These regions are associated with a cognitive control network (CCN), specifically controlling basic appetitive drives with cognitive restraint mechanisms. Conflict between appetitive responses to food and anxiety cognitions about shape, weight, eating and desire for thinness are likely to activate neural mechanisms associated with the DLPFC. An imbalanced convergence on the insular cortex by the CCN is likely to lead to a rate-limiting defect and disruption to appetitive neural responses and difficulties in interoceptive awareness in females with AN. Activation of the CCN is perhaps more prominent in women with RAN.

## Supporting Information

Table S1 List of stimuli presented during the scan: lexical descriptions of neutral images and images of high calorie foods.
(DOCX)
