# Supplementary material for: Thinking about Eating Food Activates Visual Cortex with Reduced Bilateral Cerebellar Activation in Females with Anorexia Nervosa: An fMRI Study
Source: PLoS One. 2012 Mar 27;7(3):e34000. doi: 10.1371/journal.pone.0034000 (PMC3313953; doi:10.1371/journal.pone.0034000)
Supplement: Table S1 — List of stimuli presented during the scan: lexical descriptions of neutral images and images of high calorie foods. (DOCX) [file pone.0034000.s001.docx]

**Table S1: List of images (all presented on the same-size white plate and blue background).**

| **Neutral stimuli** | **High-calorie food stimuli** |
| --- | --- |
| Some thick white twine on a roll | Almond croissant with icing sugar |
| A box of opened matches | Selection of chocolate biscuits/wafers |
| A fingernail brush | Cooked fried potato chips |
| A compass on a cord | Cream éclair with toffee coating |
| A stapler | Large cheese and salad half baguette |
| A mousetrap with cheese | Double-layer cream cake with icing |
| Two sponges | Two chocolate and toffee bars |
| Two shoe brushes | Eight chocolate biscuits |
| Two pairs of pliers | A large plate of cashew nuts |
| 12 wine corks | A large 12 piece chocolate bar |
| A spectacles case | A large plate of 'Guylian' chocolates |
| A collection of sea shells | A large deep filled bread roll |
| Five mini rolls of sellotape | Spaghetti on toast |
| Yellow clothes pegs | Tortilla chips |
| Beads for a necklace | A large plate of chocolates |
| A ball of string | A large carrot cake |
| A leather wallet | A slice of brown currant cake |
| Shoe laces | Four large chocolate fingers |
| Four hair scrunches | A large plate of “Quality Street” sweets |
| Wooden painted prongs | Battered fish, chips and peas |
| Coloured elastic bands | Toast, beans in tomato sauce, mushrooms, two fried eggs |
| A piece of rope | A large crusty bread roll with cheese |
| 3 pens | A whole thick crusted pizza |
| Tangle of string | A glazed iced bun |
| An open box of matches | Mashed potato, chicken, gravy and vegetables |
| Yellow pencils with rubbers on the end | Cheese tortilla, potato chips and dips |
| Five reels of different coloured cotton | Spaghetti bolognaise with sauce |
| A mobile phone | A large plate of pecan nuts |
| A leather wallet | A large egg custard tart |
| A yellow phone | Chips with tomato sauce |
| A sponge | A large plate of wine gums |
| A male's wallet | Chocolate bar with coconut |
| Different coloured thread | An iced pastry with cherry sauce |
| Wooden pegs | A large slice of sponge cake |
| Two yellow candles | A cheese scone |
| A used candle | A large plate of chocolate chip cookies |
| Black bulldog clips | Bananas with whipped cream and chocolate sauce |
| A red wallet | A large plate of pretzels |
| An open diary | A large plate of pasta with creamy sauce |
| Two cassette tapes | A strawberry and chocolate ice cream cone |
| A calculator | A quarter of a chocolate cake |
| Two light bulbs | Two pieces of bread with thick cheese spread |
| A pile of nails | Hash brown, two fried tomatoes, beans in tomato sauce and two fried eggs |
| An iron | A whole slice of brie cheese |
| ACD disk | Iced currant bun with a cherry |
| A hairdryer | Pasta shells with thick tomato sauce |
| Two wooden door wedges | A large plate of marsh mallows |
| A torch | A thick slice of cheesy pizza with sweetcorn |
| A chess set | A large plate of potato crisps |
| A wooden brush | A chocolate éclair |
| Brown and white heavy duty tape | A large sesame seed bun with chicken and salad filling |
| Brown and white coat hangers | A large piece of chocolate gateaux |
| Metal drawing pins | A large cheese and salad baguette |
| A crocodile clip | A snickers bar (nuts, chocolate, nougat) |
| A ball of white string | Three thick-filled chicken sandwiches |
| Coloured plastic clothes pegs | 15 chocolate digestive biscuits |
| Coloured pens | Half a thick-filled cheese and salad baguette |
| Flexi-measuring tape | A large plate of salted peanuts |
| A red wallet | A large 32 piece chocolate bar |
| A CD player | A large plate of chocolate |
| Two washing up sponges | A large plate of cheese and crackers |
| Coloured deflated balloons | A large plate of potato crisps |
| Hairdryer | A large plate of chocolate nut clusters |
| Hair comb | A chocolate and ginger cake with icing |
| Colouring pens | 12 'Kit-Kat' chocolate wafer fingers |
| Brown envelopes | A large selection of boiled sweets |
| Coloured computer disks | A plate of egg, battered fish and salad |
| Name tag keyrings | A selection of cheese, chilli, guacamole and tomato dips with nachos |
| A white computer mouse | A thick slice of pizza with cheese, mushrooms and peppers |
| Coloured paint pots with artists paint brushes | Three jam donuts with sugar coating |
| A rubber stamp | A large plate of noodles with carrots and bean sprouts |
| Swimming goggles | Jacket potato with baked beans |
| Sponge | Pasta with tomato and cheese |
| Laptop computer | A large plate of almonds |
| Calculator | A pastry tart with cherry filling |
| Hairgrips | 3 large pancakes with maple syrup |
| Tape measure | A large slice of apple pie with meringue |
| Pens | A large currant scone with whipped cream |
